# Strategies for the synthesis of the osmolyte glucosylglycerate and its precursor glycerate

**DOI:** 10.1007/s00253-024-13139-w

**Published:** 2024-04-12

**Authors:** Yentl Allaert, Arthur Leyder, Jorick Franceus, Tom Desmet

**Affiliations:** Centre for Synthetic Biology (CSB), Department of Biotechnology, Ghent University, Coupure Links 653, B-9000 Ghent, Belgium

**Keywords:** Glucosylglycerate, Glycerate, Sucrose phosphorylase, Osmolyte

## Abstract

**Abstract:**

Glycosidic osmolytes are widespread natural compounds that protect microorganisms and their macromolecules from the deleterious effects of various environmental stresses. Their protective properties have attracted considerable interest for industrial applications, especially as active ingredients in cosmetics and healthcare products. In that regard, the osmolyte glucosylglycerate is somewhat overlooked. Glucosylglycerate is typically accumulated by certain organisms when they are exposed to high salinity and nitrogen starvation, and its potent stabilizing effects have been demonstrated in vitro. However, the applications of this osmolyte have not been thoroughly explored due to the lack of a cost-efficient production process. Here, we present an overview of the progress that has been made in developing promising strategies for the synthesis of glucosylglycerate and its precursor glycerate, and discuss the remaining challenges.

**Key points:**

• *Bacterial milking could be explored for fermentative production of glucosylglycerate*

• *Glycoside phosphorylases of GH13_18 represent attractive alternatives for biocatalytic production*

• *Conversion of glycerol with alditol oxidase is a promising strategy for generating the precursor glycerate*

## Introduction

The diverse collection of natural glycosides shows countless unique bioactivities that are critical to numerous living organisms and also hold significant industrial importance (Dembitsky 2004; Elshahawi et al. 2015; Bartnik and Facey 2024). In particular, glycosidic osmolytes have properties that are interesting, yet largely unexploited in several industries. Osmolytes are highly soluble molecules that are accumulated by microorganisms to high intracellular concentrations in order to protect themselves and their macromolecules against fluctuating water activity (Becker and Wittmann 2020). This mechanism enables microbes to endure or even thrive in extreme or harsh environments.

Several glycosidic osmolytes have been identified in nature, with mannosylglycerate (2-*O*-α-mannosyl-d-glycerate, MGA) being the most extensively researched. Predominantly found in (hyper)thermophilic microorganisms, MGA not only serves as an osmoregulator in water-stressed conditions but also as a protective agent for proteins, preventing denaturation at elevated temperatures (Luley-Goedl and Nidetzky 2011). Although MGA can be produced through fermentation with a natural producer, the prohibitive production costs have hindered its widespread utilization and extensive exploration for possible applications. Despite these challenges, MGA has shown considerable potential as a penetration enhancer for cosmetic active ingredients and as a compound for the synthesis of immunostimulating agents (Schwarz 2005; Hamon et al. 2017). Glucosylglycerol (2-*O*-α-glucosylglycerol, GGO) is another notable glycosidic osmolyte, mainly found in marine cyanobacteria where it acts as the primary osmolyte in salt-stressed conditions (Hagemann 2011). Commercially known as “Glycoin” (bitop AG), the compound is obtained from sucrose and glycerol through a transglycosylation process utilizing a sucrose phosphorylase (Goedl et al. 2008). Widely incorporated into skincare products as a moisturizing agent, glucosylglycerol has been proven to promote skin elasticity, smoothness, and thickness (Harada et al. 2010).

The lesser-known glycosidic osmolyte glucosylglycerate (GGA) was originally identified in the marine cyanobacterium *Agmenellum quadruplica* but has later been observed in archaea and γ-proteobacteria as well (Kollman et al. 1979; Empadinhas and da Costa 2011). GGA plays a role in promoting osmoregulation under high salinity (Empadinhas and da Costa 2011; Nunes-Costa et al. 2017). Under nitrogen-limiting conditions, it can act as a substitute for a different negatively charged osmolyte, l-glutamate, serving as a counterion to cations like K^+^ and Na^+^ (Klähn et al. 2010). Structurally, GGA resembles both the widely known extremolyte mannosylglycerate and the commercially available glucosylglycerol, differing in the sugar moiety or in the charge of the aglycone, respectively. Because of these similarities, GGA could present an intriguing and potentially superior alternative for industrial applications. Indeed, GGA was found to be a potent protein stabilizer at elevated temperatures, during storage, or during freeze-drying operations, outperforming MGA and GGO (Faria et al. 2008; Sawangwan et al. 2010). Additionally, the sodium salt of GGA has been demonstrated to enhance collagen synthesis (Sato et al. 2014a).

Over the past few years, significant steps have been made towards achieving the efficient production of this glycoside, paving the way for its exploitation on an industrial scale. Furthermore, clear progress has been made in the cost-effective synthesis of glycerate, a necessary precursor in many of the envisaged routes for glucosylglycerate synthesis. In addition, glycerate holds potential as a valuable platform molecule for producing various other industrially relevant compounds (Wada et al. 1996; Rahman et al. 1996; Lešová et al. 2001; Fong et al. 2007; Rosseto et al. 2008). This review summarizes the diverse strategies that have been explored for synthesizing glucosylglycerate and glycerate from economical substrates, while highlighting practical considerations crucial for their large-scale production.

## Synthesis of GGA by native producers

Various microorganisms have evolved diverse metabolic pathways for the biosynthesis of GGA (Nunes-Costa et al. 2017), which can be exploited for production purposes by simulating the stressful conditions that trigger the intracellular accumulation of GGA. For instance, a twofold increase in concentration was observed in *Streptomyces caelestis* by adding 0.3% NaCl to the medium (Pospíšil et al. 2007). Various other bacteria and archaea have been reported to synthesize GGA, from the marina cyanobacterium *Synechococcus* sp. PCC 7002 to the phylopathogenic soil enterobacterium *Dickeya chrysanthemi* and the halophilic archaeon *Methanococcoides burtonii* (Kollman et al. 1979; Robertson et al. 1992; Goude et al. 2004; Empadinhas and da Costa 2011). Clearly, the identification of organisms that can act as a cell factory for the accumulation of GGA should not pose significant challenges. However, the process of isolating the accumulated GGA is far less straightforward. The compound can be extracted by cell lysis, but this approach releases all cellular contents which complicates further downstream processing. Therefore, it may be more convenient to exploit the natural efflux mechanisms for GGA, which are triggered by sudden hypoosmotic shocks (Kempf and Bremer 1998).

The process of bacterial milking, where osmolytes are harvested from native producers without disrupting the cells, was first described for the production of the compatible solute ectoine using the Gram-negative bacterium *Halomonas elongata*, and today, this process is applied on an industrial scale (Sauer and Galinski 1998). Cells grown under high-salt concentrations are harvested and resuspended in demineralized water, thus initiating a hypoosmotic shock. As a result, mechanosensitive channels in the inner membrane open, leading to the rapid release of the accumulated ectoine. After separation of biomass and solute, the bacterial cells can be recovered and a new cycle of osmolyte production can begin (Sauer and Galinski 1998). The concept of bacterial milking has also been demonstrated for the production of mannosylglycerate with a trehalose-deficient mutant of *Thermus thermophilus* RQ-1. Nearly 90% of the intracellularly accumulated MGA could be recovered, reaching a productivity of up to 0.29 g MGA L^−1^ h^−1^ (Egorova et al. 2007). Considering the structural, functional, and metabolic similarities between MGA and GGA, it may be feasible to design a similar process for the efficient production and extraction of GGA in bacterial hosts.

Although bacterial milking may be a promising method for GGA production, a few challenges are yet to be addressed. For instance, production strains should show a broad salt tolerance, and they should be able to grow well in both low- and high-salt media (Egorova et al. 2007). Furthermore, fermentation equipment should be able to withstand the corrosiveness introduced by the elevated salinity of the medium (Lang et al. 2011). Moreover, there may be a need to identify and knock out undesirable degradative metabolic pathways in the microbial producer of choice. Osmolytes like GGO and GGA were long considered to be biosynthetic endpoints, but it has recently been observed that these compatible solutes do experience significant turnover in *Synechococcus* sp. PCC 7002, suggesting that they can serve as a direct conduit toward formation of storage biopolymers (Baran et al. 2017). Additionally, glucosylglycerate hydrolases have now been identified in mycobacteria, hydrolyzing GGA to glycerate and glucose as a source of readily available energy for bacterial reactivation following nitrogen starvation (Cereija et al. 2019). If the goal is to achieve high osmolyte yields, it is essential to consider the possible presence of such degradative pathways when choosing a production strain. Finding the optimal balance between carbon- and nitrogen-sources during fermentation is also crucial, as the organic osmolyte ratio in cells depends on medium composition, where low-nitrogen media seem to favor GGA production (Goude et al. 2004). Development of a bacterial milking process for GGA production could start from natural GGA producers and increase their productivity, or alter MGA-producing strains towards GGA synthesis. Introducing a pathway for GGA synthesis in *E*. *coli* is also a viable option, as the hypoosmotic shock required for bacterial milking also leads to the rapid release of solutes in the medium (Tsapis and Kepes 1977).

## Phosphorylase-catalyzed synthesis of GGA

As an alternative to bacterial milking, the use of isolated enzymes has been explored for the biocatalytic production of osmolyte. Indeed, the success story of the large-scale phosphorylase-catalyzed synthesis of GGO has sparked interest in the development of a similar process for GGA. Several relevant enzymes for this purpose can be found in subfamily 18 of glycoside hydrolase family 13 (GH13_18) of the carbohydrate-active enzyme (CAZy) database (Drula et al. 2022). The most famous representative of this family is sucrose phosphorylase (SP, EC 2.4.1.7). This enzyme catalyzes the reversible phosphorolysis of sucrose, but it can also be applied as a versatile and efficient transglycosylase in vitro (Franceus and Desmet 2020). Its preferred glycosyl donor substrate sucrose is highly reactive, but far less expensive than the activated sugars that are typically required by glucosyltransferases (e.g., UDP-glucose). In addition, the remarkable promiscuity of SP facilitates the transfer of the glucosyl moiety to a wide range of glycosyl acceptor substrates other than inorganic phosphate (Aerts et al. 2011).

**Fig. 1:**
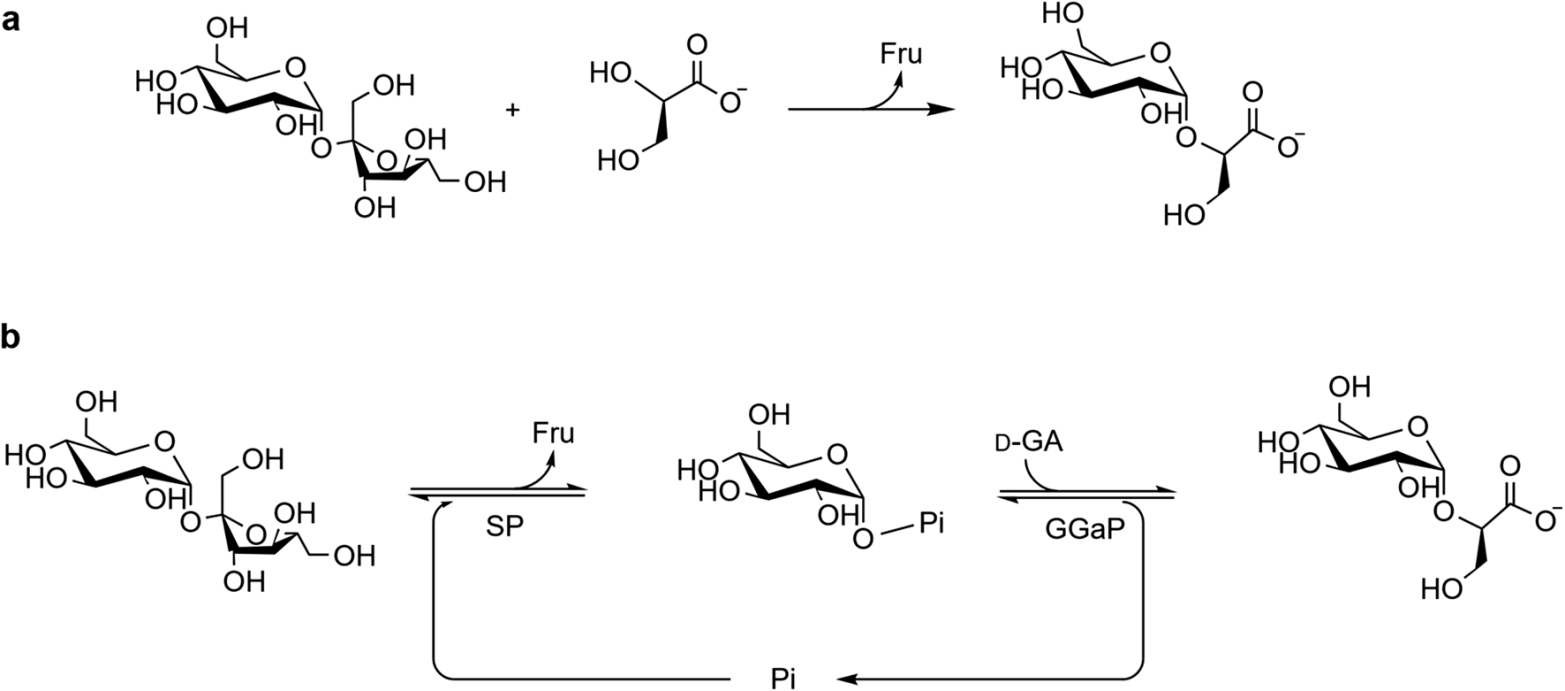
Reactions catalyzed by phosphorylases for the synthesis of 2-*O*-α-glucosyl-d-glycerate. **a** One-step transglucosylation performed by *Lm*SP or *Xp*GP, **b** cascade reaction with SP and GGaP, where glucose 1-phosphate acts as intermediate. (Fru: fructose, Pi: inorganic phosphate, SP: sucrose phosphorylase, GGaP: glucosylglycerate phosphorylase: d-GA: d-glycerate)

The first biocatalytic route to be proposed for the synthesis of GGA was a one-step process involving the transglycosylation activity of the sucrose phosphorylase from *Leuconostoc mesenteroides* (*Lm*SP) (Fig. 1a) (Sawangwan et al. 2009). In this process, the glucosyl moiety of sucrose is selectively transferred to the 2-*O*-position of glycerate. Both d-glycerate and l-glycerate can be used as acceptor, although the former is preferred by *Lm*SP and resulted in a yield of 59% (Sawangwan et al. 2009). However, since this process is based on a promiscuous side activity of SP, the reaction is inherently slow and also suffers from considerable kinetic competition between glucosyl transfer to glycerate (transglycosylation) and glycosyl transfer to water (hydrolysis). Consequently, the reaction conditions must be carefully optimized to favor GGA synthesis. Using *Lm*SP, a 2.5-fold molar excess of donor over acceptor and a saturating level of d-glycerate (300 mM) need to be supplied to achieve a product yield of 91%. Nevertheless, extended reaction times of up to 72 h are still required (Sawangwan et al. 2009).

It was later discovered that the same CAZy subfamily (GH13_18) also contains strict glucosylglycerate phosphorylases (GGaP, EC 2.4.1.352). These enzymes are not active on sucrose, but exclusively catalyze the reversible phosphorolysis of 2-*O*-α-d-glucosylglycerate to α-glucose 1-phosphate (Glc1P) and d-glycerate (Franceus et al. 2017). This novel specificity was identified after it had been observed that genes encoding putative SP homologs tend to be located near genes involved in GGA metabolism (Empadinhas and da Costa 2011; Nunes-Costa et al. 2017). Currently characterized GGaPs originate from *Allomeiothermus silvanus*, *Spirochaeta thermophila*, and *Escherichia coli*, and all are known to show very high affinity for d-glycerate compared to other phosphorylases within the GH13_18 subfamily, with reported *K*_M_ values between 1 and 5 mM (Table 1) (Franceus 2017, Mukherjee 2018). Additionally, the specific activity of GGaP towards d-glycerate (110 U/mg) is far higher than that of SPs (< 1 U/mg) (Franceus et al. 2017). These properties present the opportunity to develop a more efficient alternative biocatalytic process with a considerably improved space–time yield compared to the one-step transglycosylation process using *Lm*SP (Sawangwan et al. 2009). An evident drawback is the need for Glc1P instead of sucrose as the glucosyl donor, but this substrate can be generated in situ from sucrose by the native activity of SP, or from a different bulk sugar by the action of a different phosphorylase (Fig. 1b). Similar one-pot cascade processes have already been developed for the production of cellobiose, trehalose, and GGO (Taniguchi et al. 1991; Schwarz et al. 2007; Zhang et al. 2020). After careful selection of the most attractive enzymes and optimization of reaction conditions, a comparable set-up for the production of GGO by the combined action of *Lm*SP and a strict glucosylglycerol phosphorylase resulted in a yield of 89% with a titer of 1.78 M and a productivity of 24.3 g/L/h (Zhang et al. 2020). It would be interesting to perform similar efforts for the coupled two-step production of GGA.

**Table 1:** Reported kinetic parameters and process performance parameters for various glycoside phosphorylases from GH13_18

Enzyme	K_M, sucrose_ (mM)	K_M, d-GA_ (mM)	Specific activity (U/mg)	STY (mMh^−1^)	*v*_acceptor_/*v*_water_^a^
SP	0.8–14^d^	175–350^e^	< 1^e^	3.8^b,f^	0.5–1.4^d^
GGaP^e^	-	1–5	110	n.a	1100
*Xp*GP^g^	67	20	53	73.5 ^c^	> 10

*SP* Sucrose phosphorylase; *GGaP* strict glucosylglycerate phosphorylase; *XpGP* bifunctional sucrose-active glucosylglycerate phosphorylase from *Xylanomonas proteatiae*; *GA* glycerate; *STY* space–time yield; *v* specific activity; *n.a.* not available. ^a^Ratio between specific activity on d-glycerate and the hydrolytic activity in the absence of d-glycerate at pH 7.0 and 37 °C or 30 °C for SPs and *Xp*GP, respectively. ^b^72 h-reaction with 5.5 μM purified enzyme, 300 mM d-glycerate, and 800 mM sucrose at 30 °C and pH 7.0. ^c^4 h-reaction with 3.7 μM purified enzyme, 300 mM d-glycerate, and 400 mM sucrose at 30 °C and pH 7.0. ^d^(Aerts et al. 2011); ^e^(Franceus et al. 2017); ^f^(Sawangwan et al. 2009); ^g^(Franceus et al. 2024)

Recently, a third type of phosphorylase capable of synthesizing GGA was discovered by further searching subfamily GH13_18 for novel substrate specificities (Franceus et al. 2024). Indeed, the phosphorylase from *Xylanomonas proteatiae* (*Xp*GP) showed very high activity on d-glycerate as acceptor (ratio of transglycosylation over hydrolysis > 10), but unlike the strict GGaPs, it was still able to use sucrose as donor substrate, albeit with lower affinity than true SPs (Table 1) (Franceus et al. 2024). This sucrose-active glucosylglycerate phosphorylase thus seems to combine the useful acceptor promiscuity of *Lm*SP with the excellent catalytic efficiency for glycerate of GGaP. Therefore, it is a highly promising biocatalyst for a one-step process for the synthesis of GGA from sucrose and glycerate (Fig. 1a). While the equivalent transglycosylation process with *Lm*SP strongly suffers from substrate loss through hydrolysis, this undesired side activity can effectively be suppressed in the process with *Xp*GP. Just a 1.33-fold molar excess of sucrose was found to be sufficient to achieve yields up to 98%. In addition, incubation times could significantly be reduced due to a much higher reaction rate. While reactions with 5 μM *Xp*GP and equimolar substrate concentrations reached maximal conversion after approximately 2 h, only 3% of the sucrose was converted at that time in the equivalent reaction with *Lm*SP (Franceus et al. 2024).

## Production of d-glycerate as precursor for GGA synthesis

### Synthesis of d-glycerate from glycerol

The feasibility of scaling up the phosphorylase-catalyzed synthesis of GGA is closely tied to the commercial availability of the required precursor d-glycerate. Several strategies have been explored to generate d-glycerate from the cheap bulk substrate glycerol, which is a major by-product (10 wt%) of biodiesel production. Glycerol is an attractive feedstock for further valorization due to its high abundance and low price (Chen and Liu 2016; Kaur et al. 2020). Glycerol can be chemically oxidized to glyceric acid with the use of supported metal nanoparticle catalysts such as Pd, Pt, or gold catalysts, but these processes suffer from a few considerable drawbacks (Carrettin et al. 2003; Villa et al. 2010, 2015). First, achieving selective oxidation is challenging due to the comparable reactivity of all three hydroxyl groups (Pagliaro et al. 2007). Without thorough reaction control, glyceric acid is further oxidized to the undesired byproducts tartronic or mesoxalic acid. Second, the requirement of the precious metal catalysts has a significant environmental impact (Zhang et al. 2021). Third, these processes result in the formation of racemic glyceric acid. As an intermediate for GGA synthesis, enantiopure d-glycerate would be the desired oxidation product, not only because it is required to obtain the natural isomer of the osmolyte (i.e., 2-*O*-α-glucosyl-d-glycerate), but also because d-glycerate tends to be preferred over l-glycerate as glycosyl acceptor by the phosphorylase that performs the desired transglycosylation reactions. Chiral resolution by separation methods such as chromatography or crystallization may be considered (Teng et al. 2022). However, the use of these additional expensive processes for low-cost applications seems unrealistic from an economic perspective.

**Fig. 2:**
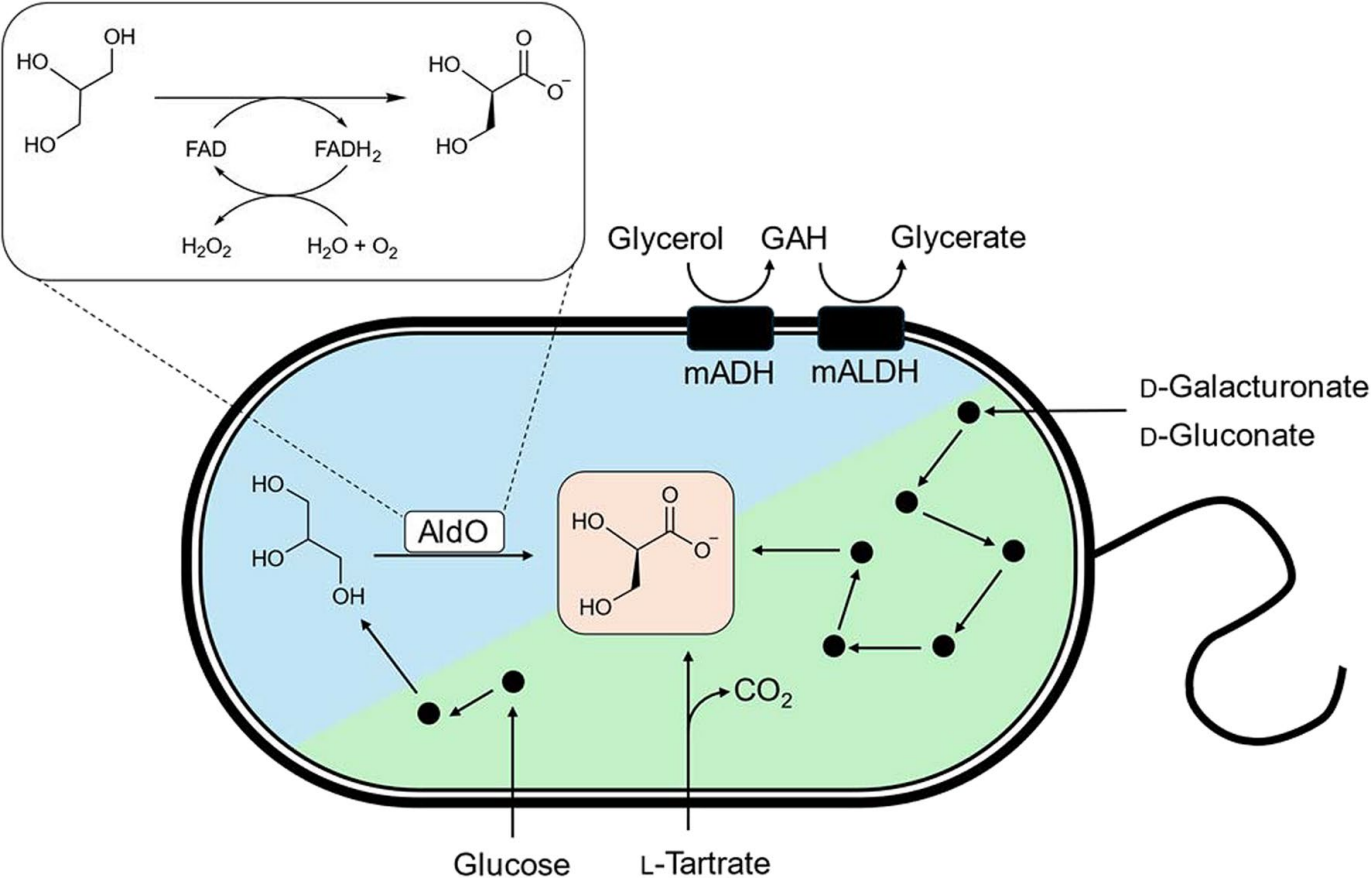
Schematic representation of the several biotechnological routes for d-glycerate synthesis. Blue denotes the strategies based on the valorization of glycerol, while green indicates approaches based on alternative substrates. (AldO: alditol oxidase, mADH: membrane-bound alcohol dehydrogenase, mALDH: membrane-bound aldehyde dehydrogenase, GAH: glyceraldehyde, FAD: flavin adenine dinucleotide)

Biotechnology can offer a solution to the challenges associated with chemical oxidation processes (Fig. 2). Such processes produce less waste are operated under mild reaction conditions, and their selectivity minimizes the need for additional expensive separation processes (Sheldon and Woodley 2018). For instance, acetic acid bacteria (AAB) can oxidize various alcohols (e.g., ethanol, glycerol, and d-sorbitol) through a process known as oxidative fermentation facilitated by membrane-bound dehydrogenases. Afterwards, the oxidative products are released in the culture medium, which facilitates the downstream processing (Saichana et al. 2015). When glycerol is provided, this alcohol is first converted to glyceraldehyde via membrane-bound alcohol dehydrogenase (mADH, EC 1.1.99.8). Subsequently, glyceraldehyde can be further oxidized to glycerate by membrane-bound aldehyde dehydrogenase (mALDH, EC 1.2.99.3) (Habe et al. 2009, 2010). Some mADHs are more efficient at selectively forming one enantiomer of glycerate. For example, *Acetobacter tropicalis* NBRC16470 was found to produce d-glycerate with near-perfect purity (> 99%), whereas fed-batch fermentation with *Gluconobacter frateurii* NBRC103465 resulted in higher product titer with a more modest enantiomeric excess of 72% (Habe et al. 2009). Unfortunately, the mechanisms determining the enantiomeric composition of the produced glycerate remain unknown (Habe et al. 2010). In addition, methanol, present in raw glycerol, has significant inhibitory effects on mADH, even at high glycerol concentrations (Sato et al. 2013). Consequently, *G. frateurii* has been engineered to become methanol-resistant (Sato et al. 2014b) and methylotrophic AAB have been explored as potential glycerate-producing strains (Sato et al. 2017). While mADHs have been intensively studied in AAB (Yakushi and Matsushita 2010), little is known about the contribution of mALDH to the synthesis of glycerate.

Besides fermentation, biocatalytic production with isolated enzymes is also a promising option. Oxidases are in that regard more attractive than dehydrogenases since they rely on oxygen as electron acceptor instead of NAD(P)^+^, making it possible to use them also in cell-free systems without the need for expensive cofactors that either need to be supplied in stoichiometric amounts, or need to be regenerated in situ (Wahart et al. 2022). Unfortunately, an oxidase that specifically acts on glycerol has not yet been discovered. However, alditol oxidase (AldO, EC 1.1.3.41) can oxidize glycerol in a highly regio- and enantioselective manner. While its native substrates are longer polyols (e.g., xylitol, sorbitol), the enzyme displays promiscuous activities on glycerol and aliphatic or aromatic 1,2-diols, albeit with poor catalytic efficiency (van Hellemond et al. 2009). Depending on the specific substrate, AldO performs the selective oxidation of the primary hydroxyl group of its substrates to produce either the corresponding α-hydroxy aldehyde or α-hydroxy acid (van Hellemond et al. 2009). To that end, a hydride is transferred from the primary carbon atom to a flavin adenine dinucleotide (FAD) cofactor that is co-expressed with and covalently bound to the enzyme, after which molecular oxygen acts as the final electron acceptor, producing hydrogen peroxide as by-product. During this two-step oxidation, d-glycerate rather than d-glyceraldehyde is generated as final product.

The first characterized alditol oxidase originated from the actinomycete *Streptomyces coelicolor* A3(2) (*Sc*AldO) (Heuts et al. 2007). Although the enzyme is relatively stable, with a half-life of 5 h at 50 °C (van Hellemond et al. 2009), efforts were made to search for more thermostable homologs that are preferred by industry since operating at elevated temperatures increases the reaction rate and minimize microbial contamination (Suresh et al. 2021). Through genome mining, a homolog of *Sc*AldO was discovered in the genome of the thermophilic bacterium *Acidothermus cellulolyticus* 11B (*Ac*AldO), an organism isolated from acidic hot springs (Winter et al. 2012). Recently, several thermostable alditol oxidases have been characterized, and their activity on glycerol has been compared (Santema et al. 2024). The homolog from *Thermopolyspora flexuosa* (*Tf*AldO) was found to be a particularly promising candidate due to its favorable kinetic properties (Chen et al. 2022).

The potential of an AldO-catalyzed process for the synthesis of d-glycerate was first demonstrated by Gerstenbruch et al., yielding 2.0 g/L d-glycerate (99.6% ee) after 60 h using resting whole cells in which *Sc*AldO was overexpressed (Gerstenbruch et al. 2012). These results have fuelled the development of improved variants of the enzyme (Table 2). Although initial attempts to rationally engineer *Sc*AldO by combinatorial saturation mutagenesis of active site residues were unsuccessful, a random mutagenesis effort by error-prone PCR uncovered a quadruple mutant (V125M/A244T/V133M/G399R) that showed a modest 2.4-fold improvement compared to the wild-type enzyme (Gerstenbruch et al. 2012). The variant underwent further engineering with additional rounds of error-prone PCR and synthetic shuffling, resulting in an additional 11 mutations and 1.3-fold higher catalytic efficiency. Upon introducing this mutant and removing the genes involved in the phosphorylation and oxidation of d-glycerate, 30.1 g/L was accumulated in *E*. *coli* after 70-h fermentation (Zhang et al. 2021). It was however striking that the glycerol to d-glycerate yield was much lower than the theoretical yield (0.376 to 1 mol/mol). Instead of completing the second step of glycerol oxidation due to the lower affinity towards the intermediate product, glyceraldehyde could be metabolized by other enzymes or could enter the central carbon metabolism of *E. coli*. In a different study, *Sc*AldO was engineered through a process of controlled continuous evolution (Rosenthal et al. 2023). Following in vivo gene diversification, individual bacteria exhibiting improved oxidase activity, quantified by an increase in fluorescence signals, were isolated using a microfluidics device. This iterative process was repeated multiple times, with the initial rounds displaying a prevalence of the F274Y mutation. However, this mutation was later outcompeted by the F278C mutation (10.5-fold increase in catalytic efficiency) which dominated the population within days. Finally, in the most recent engineering study, in silico mutagenesis and analysis of 50 single and double mutants of *Tf*AldO resulted in 8 putative improved variants that were validated experimentally. The V258L/P259I mutations were found to cause a threefold increase in catalytic efficiency, primarily attributed to an improvement in *k*_*cat*_ (Santema et al. 2024).

**Table 2:** Kinetic properties of wild-type and engineered alditol oxidases

Enzyme	Mutations	K_M_ (mM)	*k*_*cat*_ (s^−1^)	*k*_*cat*_*/*K_M_ (s^−1^ M^−1^)	Reference
*Sc*AldO	Wild-type	350	1.6	4.6	(van Hellemond et al. 2009)
*Sc*AldO	V125M, V133M, A244T, G339R	110	0.72	5.1	(Gerstenbruch et al. 2012)
*Sc*AldO	R22C, D27A, S68G, A75V, V125M, S129P, V133M, V148T, R232Q, A244T, F262L, E310D, G339R, A368G, E383R	66.91	0.57	8.5	(Zhang et al. 2021)
*Sc*AldO	F278C	49.95	7.81	156.4	(Rosenthal et al. 2023)
*Ac*AldO	Wild-type	270	1.3	4.8	(Winter et al. 2012)
*Tf*AldO	Wild-type	50	1.6	32	(Santema et al. 2024)
*Tf*AldO	V258L, P259I	41	4.0	98	(Santema et al. 2024)

*Sc*AldO Alditol oxidase from Streptomyces coelicolor A3(2); *Ac*AldO alditol oxidase from Acidothermus cellulolyticus 11B; *Tf*AldO alditol oxidase from Thermopolyspora flexuosa

### Biotechnological synthesis of d-glycerate from other substrates

Studies on the biotechnological synthesis of d-glycerate are often framed in the context of valorizing crude glycerol derived from biodiesel production. However, glycerol can also be obtained from glucose using engineered *E*. *coli*. Glycerol is not only tied to carbon stress response in *E. coli*, but it is also less efficiently utilized than glucose, a common carbon source for fermentation (Martínez-Gómez et al. 2012). While the in vivo generation of glycerol by heterologous overexpression of two *Saccharomyces cerevisiae* genes and subsequent oxidation by AldO resulted in the highest specific productivity of glycerate in *E. coli* to date (1.72-g glycerate/g cells) (Long et al. 2023), the formation of lactate, pyruvate, and acetate poses difficulties in the purification of the desired product. In addition, attempts to produce glycerate from substrates other than glycerol have been reported. l-Tartrate, generated during wine fermentation, can efficiently get decarboxylated to d-glycerate via l-tartrate decarboxylase from *Pseudomonas* sp. with molar yield of nearly 100% and an enantiomeric excess of 92% (Furuyoshi et al. 1989, 1991). Furthermore, advances in synthetic biology have resulted in the construction of novel metabolic pathways that enable d-glycerate synthesis. Engineered *E. coli* could convert d-galacturonate to optical pure d-glycerate with a titer of 4.8 g/L and a molar yield of 83% (Fox and Prather 2020). The pathway was further extended to cope with d-gluconate as starting substrate or a mixture of both. Interestingly, the strain could consistently produce d-glycerate across the range of any mixed substrate feed (Ni and Prather 2024).

## Conclusion and perspectives

Various routes for the synthesis of glucosylglycerate have been explored and refined over the years, steadily advancing us towards the realization of a cost-effective process that would enable this attractive osmolyte to be produced on an industrial scale. The recent discovery of a bifunctional phosphorylase that is capable of efficiently synthesizing glucosylglycerate from sucrose and glycerate may be particularly promising in that regard, especially when combined with engineered alditol oxidases that can readily generate the required glycosyl acceptor from glycerol. Although such a biocatalytic cascade may already be feasible, either as a one-pot dual-enzyme system or as two separate but subsequent conversions, it is clear that further research is necessary to optimize both the process and the enzymes. First, the dependence of oxidases on O_2_ presents a few notorious challenges related to the low transfer rate of O_2_ from the gas to the aqueous phase, the low solubility of O_2_, the energy required to actively supply O_2_ to the medium by shaking or stirring and the resulting destabilizing effect on the enzymes (Al-Shameri et al. 2023). Second, the phosphorylase and best-performing oxidase display different preferences in pH and reaction temperature, which limits their compatibility within one elegant system. Third, although the kinetic parameters of the designed glycerol oxidases are already appealing, there is room for further improvement. It seems that an integrated approach involving process and protein engineering may be most appropriate to address these problems.

## References

[CR1] Aerts D, Verhaeghe TF, Roman BI, Stevens CV, Desmet T, Soetaert W (2011) Transglucosylation potential of six sucrose phosphorylases toward different classes of acceptors. Carbohydr Res 346:1860–1867. 10.1016/j.carres.2011.06.024 21798524 10.1016/j.carres.2011.06.024

[CR2] Al-Shameri A, Schmermund L, Sieber V (2023) Engineering approaches for O_2_-dependent enzymes. Curr Opin Green Sustain Chem 40:100733. 10.1016/j.cogsc.2022.100733

[CR3] Baran R, Lau R, Bowen BP, Diamond S, Jose N, Garcia-Pichel F, Northen TR (2017) Extensive turnover of compatible solutes in cyanobacteria revealed by deuterium oxide (D_2_O) stable isotope probing. ACS Chem Biol 12:674–681. 10.1021/acschembio.6b00890 28068058 10.1021/acschembio.6b00890

[CR4] Bartnik M, Facey P (2024) Chapter 7 - glycosides. In: McCreath SB, Clement YN (eds) Pharmacognosy (Second Edition). Academic Press, pp 103–165

[CR5] Becker J, Wittmann C (2020) Microbial production of extremolytes — high-value active ingredients for nutrition, health care, and well-being. Curr Opin Biotechnol 65:118–128. 10.1016/j.copbio.2020.02.010 32199140 10.1016/j.copbio.2020.02.010

[CR6] Carrettin S, McMorn P, Johnston P, Griffin K, Kiely CJ, Hutchings GJ (2003) Oxidation of glycerol using supported Pt, Pd and Au catalysts. Phys Chem Chem Phys 5:1329–1336. 10.1039/B212047J

[CR7] Cereija TB, Alarico S, Lourenço EC, Manso JA, Ventura MR, Empadinhas N, Macedo-Ribeiro S, Pereira PJB (2019) The structural characterization of a glucosylglycerate hydrolase provides insights into the molecular mechanism of mycobacterial recovery from nitrogen starvation. IUCrJ 6:572–585. 10.1107/S2052252519005372 31316802 10.1107/S2052252519005372 PMC6608630

[CR8] Chen Z, Liu D (2016) Toward glycerol biorefinery: metabolic engineering for the production of biofuels and chemicals from glycerol. Biotechnol Biofuels 9:205. 10.1186/s13068-016-0625-8 27729943 10.1186/s13068-016-0625-8 PMC5048440

[CR9] Chen Z, Fei K, Hu Y, Xu X, Gao X-D, Li Z (2022) Identification of a novel alditol oxidase from *Thermopolyspora flexuosa* with potential application in D-glyceric acid production. Mol Biotechnol 64:804–813. 10.1007/s12033-022-00459-3 35129810 10.1007/s12033-022-00459-3

[CR10] Dembitsky VM (2004) Chemistry and biodiversity of the biologically active natural glycosides. Chem Biodivers 1:673–781 17191879 10.1002/cbdv.200490060

[CR11] Drula E, Garron M-L, Dogan S, Lombard V, Henrissat B, Terrapon N (2022) The carbohydrate-active enzyme database: functions and literature. Nucleic Acids Res 50:D571–D577. 10.1093/nar/gkab1045 34850161 10.1093/nar/gkab1045 PMC8728194

[CR12] Egorova K, Grudieva T, Morinez C, Kube J, Santos H, da Costa MS, Antranikian G (2007) High yield of mannosylglycerate production by upshock fermentation and bacterial milking of trehalose-deficient mutant *Thermus thermophilus* RQ-1. Appl Microbiol Biotechnol 75:1039–1045. 10.1007/s00253-007-0915-y 17361428 10.1007/s00253-007-0915-y

[CR13] Elshahawi SI, Shaaban KA, Kharel MK, Thorson JS (2015) A comprehensive review of glycosylated bacterial natural products. Chem Soc Rev 44:7591–7697. 10.1039/C4CS00426D 25735878 10.1039/c4cs00426d PMC4560691

[CR14] Empadinhas N, da Costa MS (2011) Diversity, biological roles and biosynthetic pathways for sugar-glycerate containing compatible solutes in bacteria and archaea. Environ Microbiol 13:2056–2077. 10.1111/j.1462-2920.2010.02390.x 21176052 10.1111/j.1462-2920.2010.02390.x

[CR15] Faria TQ, Mingote A, Siopa F, Ventura R, Maycock C, Santos H (2008) Design of new enzyme stabilizers inspired by glycosides of hyperthermophilic microorganisms. Carbohydr Res 343:3025–3033. 10.1016/j.carres.2008.08.030 18822412 10.1016/j.carres.2008.08.030

[CR16] Fong C, Wells D, Krodkiewska I, Booth J, Hartley PG (2007) Synthesis and mesophases of glycerate surfactants. J Phys Chem B 111:1384–1392. 10.1021/jp0659655 17286353 10.1021/jp0659655

[CR17] Fox KJ, Prather KLJ (2020) Production of d-glyceric acid from D-galacturonate in *Escherichia coli*. J Ind Microbiol Biotechnol 47:1075–1081. 10.1007/s10295-020-02323-2 33057913 10.1007/s10295-020-02323-2

[CR18] Franceus J, Desmet T (2020) Sucrose phosphorylase and related enzymes in glycoside hydrolase family 13: discovery, application and engineering. Int J Mol Sci 21:2526. 10.3390/ijms21072526 32260541 10.3390/ijms21072526 PMC7178133

[CR19] Franceus J, Pinel D, Desmet T (2017) Glucosylglycerate phosphorylase, an enzyme with novel specificity involved in compatible solute metabolism. Appl Environ Microbiol 83:e01434-e1517. 10.1128/AEM.01434-17 28754708 10.1128/AEM.01434-17 PMC5601347

[CR20] Franceus J, Steynen M, Allaert Y, Bredael K, D’hooghe M, Desmet T (2024) High-yield synthesis of 2-*O*-α-D-glucosyl-D-glycerate by a bifunctional glycoside phosphorylase. Appl Microbiol Biotechnol 108:1–9. 10.1007/s00253-023-12970-x 38175244 10.1007/s00253-023-12970-x PMC13498057

[CR21] Furuyoshi S, Kawabata N, Tanaka H, Soda K (1989) Enzymatic production of D-glycerate from L-tartrate. Agric Biol Chem 53:2101–2105. 10.1080/00021369.1989.10869643

[CR22] Furuyoshi S, Nawa Y, Kawabata N, Tanaka H, Soda K (1991) Purification and characterization of a new NAD^+^-dependent enzyme, L-tartrate decarboxylase, from *Pseudomonas* sp. group Ve-2. J Biochem (tokyo) 110:520–525. 10.1093/oxfordjournals.jbchem.a123613 1778975 10.1093/oxfordjournals.jbchem.a123613

[CR23] Gerstenbruch S, Wulf H, Mußmann N, O’Connell T, Maurer K-H, Bornscheuer UT (2012) Asymmetric synthesis of D-glyceric acid by an alditol oxidase and directed evolution for enhanced oxidative activity towards glycerol. Appl Microbiol Biotechnol 96:1243–1252. 10.1007/s00253-012-3885-7 22290646 10.1007/s00253-012-3885-7

[CR24] Goedl C, Sawangwan T, Mueller M, Schwarz A, Nidetzky B (2008) A high-yielding biocatalytic process for the production of 2-*O*-(α-D-glucopyranosyl)-sn-glycerol, a natural osmolyte and useful moisturizing ingredient. Angew Chem Int Ed 47:10086–10089. 10.1002/anie.200803562 10.1002/anie.200803562 19025748

[CR25] Goude R, Renaud S, Bonnassie S, Bernard T, Blanco C (2004) Glutamine, glutamate, and α-glucosylglycerate are the major osmotic solutes accumulated by *Erwinia chrysanthemi* strain 3937. Appl Environ Microbiol 70:6535–6541. 10.1128/AEM.70.11.6535-6541.2004 15528516 10.1128/AEM.70.11.6535-6541.2004 PMC525223

[CR26] Habe H, Shimada Y, Yakushi T, Hattori H, Ano Y, Fukuoka T, Kitamoto D, Itagaki M, Watanabe K, Yanagishita H, Matsushita K, Sakaki K (2009) Microbial production of glyceric acid, an organic acid that can be mass produced from glycerol. Appl Environ Microbiol 75:7760–7766. 10.1128/AEM.01535-09 19837846 10.1128/AEM.01535-09 PMC2794115

[CR27] Habe H, Shimada Y, Fukuoka T, Kitamoto D, Itagaki M, Watanabe K, Yanagishita H, Yakushi T, Matsushita K, Sakaki K (2010) Use of a *Gluconobacter frateurii* mutant to prevent dihydroxyacetone accumulation during glyceric acid production from glycerol. Biosci Biotechnol Biochem 74:2330–2332. 10.1271/bbb.100406 21071844 10.1271/bbb.100406

[CR28] Hagemann M (2011) Molecular biology of cyanobacterial salt acclimation. FEMS Microbiol Rev 35:87–123. 10.1111/j.1574-6976.2010.00234.x 20618868 10.1111/j.1574-6976.2010.00234.x

[CR29] Hamon N, Mouline CC, Travert M (2017) Synthesis of mannosylglycerate derivatives as immunostimulating agents. Eur J Org Chem 2017:4803–4819. 10.1002/ejoc.201700682

[CR30] Harada N, Zhao J, Kurihara H, Nagata N, Okajima K (2010) Effects of topical application of α-D-Glucosylglycerol on dermal levels of insulin-like growth Factor-I in mice and on facial skin elasticity in humans. Biosci Biotechnol Biochem 74:759–765. 10.1271/bbb.90797 20378988 10.1271/bbb.90797

[CR31] Heuts DPHM, van Hellemond EW, Janssen DB, Fraaije MW (2007) Discovery, characterization, and kinetic analysis of an alditol oxidase from *Streptomyces coelicolor**. J Biol Chem 282:20283–20291. 10.1074/jbc.M610849200 17517896 10.1074/jbc.M610849200

[CR32] Kaur J, Sarma AK, Jha MK, Gera P (2020) Valorisation of crude glycerol to value-added products: perspectives of process technology, economics and environmental issues. Biotechnol Rep 27:e00487. 10.1016/j.btre.2020.e00487 10.1016/j.btre.2020.e00487 PMC7334398 32642454

[CR33] Kempf B, Bremer E (1998) Uptake and synthesis of compatible solutes as microbial stress responses to high-osmolality environments. Arch Microbiol 170:319–330. 10.1007/s002030050649 9818351 10.1007/s002030050649

[CR34] Klähn S, Steglich C, Hess WR, Hagemann M (2010) Glucosylglycerate: a secondary compatible solute common to marine cyanobacteria from nitrogen-poor environments. Environ Microbiol 12:83–94. 10.1111/j.1462-2920.2009.02045.x 19735283 10.1111/j.1462-2920.2009.02045.x

[CR35] Kollman VH, Hanners JL, London RE, Adame EG, Walker TE (1979) Photosynthetic preparation and characterization of 13C-labeled carbohydrates in agmenellum quadruplicatum. Carbohydr Res 73:193–202. 10.1016/S0008-6215(00)85489-0

[CR36] Lang Y, Bai L, Ren Y, Zhang L, Nagata S (2011) Production of ectoine through a combined process that uses both growing and resting cells of *Halomonas salina* DSM 5928T. Extremophiles 15:303–310. 10.1007/s00792-011-0360-9 21331633 10.1007/s00792-011-0360-9

[CR37] Lešová K, Šturdíková M, Proksa B, Pigoš M, Liptaj T (2001) OR-1—a mixture of esters of glyceric acid produced by *Penicillium funiculosum* and its antitrypsin activity. Folia Microbiol (praha) 46:21–23. 10.1007/BF02825878 11501469 10.1007/BF02825878

[CR38] Long BHD, Matsubara K, Tanaka T, Ohara H, Aso Y (2023) Production of glycerate from glucose using engineered *Escherichia coli*. J Biosci Bioeng 135:375–381. 10.1016/j.jbiosc.2023.02.002 36841726 10.1016/j.jbiosc.2023.02.002

[CR39] Luley-Goedl C, Nidetzky B (2011) Glycosides as compatible solutes: biosynthesis and applications. Nat Prod Rep 28:875–896. 10.1039/C0NP00067A 21390397 10.1039/c0np00067a

[CR40] Martínez-Gómez K, Flores N, Castañeda HM, Martínez-Batallar G, Hernández-Chávez G, Ramírez OT, Gosset G, Encarnación S, Bolivar F (2012) New insights into *Escherichia coli* metabolism: carbon scavenging, acetate metabolism and carbon recycling responses during growth on glycerol. Microb Cell Factories 11:46. 10.1186/1475-2859-11-46 10.1186/1475-2859-11-46 PMC3390287 22513097

[CR41] Ni C, Prather KLJ (2024) Consistent biosynthesis of D-glycerate from variable mixed substrates. Metab Eng. 10.1016/j.ymben.2024.01.001 38185463 10.1016/j.ymben.2024.01.001

[CR42] Nunes-Costa D, Maranha A, Costa M, Alarico S, Empadinhas N (2017) Glucosylglycerate metabolism, bioversatility and mycobacterial survival. Glycobiology 27:213–227. 10.1093/glycob/cww132 28025249 10.1093/glycob/cww132

[CR43] Pagliaro M, Ciriminna R, Kimura H, Rossi M, Della Pina C (2007) From glycerol to value-added products. Angew Chem Int Ed 46:4434–4440. 10.1002/anie.200604694 10.1002/anie.200604694 17471485

[CR44] Pospíšil S, Halada P, Petříček M, Sedmera P (2007) Glucosylglycerate is an osmotic solute and an extracellular metabolite produced by *Streptomyces caelestis*. Folia Microbiol (praha) 52:451–456. 10.1007/BF02932103 18298040 10.1007/BF02932103

[CR45] Rahman MA, Humphreys RW, Wu SR (1996) Biodegradable fabric conditioning molecules based on glyceric acid. U.S. Patent No. 5,500,139. Washington, DC: U.S. Patent and Trademark Office

[CR46] Robertson DE, Lai M-C, Gunsalus RP, Roberts MF (1992) Composition, variation, and dynamics of major osmotic solutes in *Methanohalophilus* strain FDF1. Appl Environ Microbiol 58:2438–2443. 10.1128/aem.58.8.2438-2443.1992 16348748 10.1128/aem.58.8.2438-2443.1992 PMC195800

[CR47] Rosenthal RG, Diana Zhang X, Đurđić KI, Collins JJ, Weitz DA (2023) Controlled continuous evolution of enzymatic activity screened at ultrahigh throughput using drop-based microfluidics. Angew Chem 135:e202303112. 10.1002/ange.202303112 10.1002/anie.202303112 37019845

[CR48] Rosseto R, Tcacenco CM, Ranganathan R, Hajdu J (2008) Synthesis of phosphatidylcholine analogues derived from glyceric acid: a new class of biologically active phospholipid compounds. Tetrahedron Lett 49:3500–3503. 10.1016/j.tetlet.2008.03.084 19844592 10.1016/j.tetlet.2008.03.084 PMC2763321

[CR49] Saichana N, Matsushita K, Adachi O, Frébort I, Frebortova J (2015) Acetic acid bacteria: a group of bacteria with versatile biotechnological applications. Biotechnol Adv 33:1260–1271. 10.1016/j.biotechadv.2014.12.001 25485864 10.1016/j.biotechadv.2014.12.001

[CR50] Santema LL, Rotilio L, Xiang R, Tjallinks G, Guallar V, Mattevi A, Fraaije MW (2024) Discovery and biochemical characterization of thermostable glycerol oxidases. Appl Microbiol Biotechnol 108:1–14. 10.1007/s00253-023-12883-9 38183484 10.1007/s00253-023-12883-9 PMC10771423

[CR51] Sato S, Morita N, Kitamoto D, Yakushi T, Matsushita K, Habe H (2013) Change in product selectivity during the production of glyceric acid from glycerol by *Gluconobacter* strains in the presence of methanol. AMB Express 3:20. 10.1186/2191-0855-3-20 23547945 10.1186/2191-0855-3-20 PMC3627625

[CR52] Sato S, Kitamoto D, Habe H (2014a) In vitro evaluation of glyceric acid and its glucosyl derivative, α-glucosylglyceric acid, as cell proliferation inducers and protective solutes. Biosci Biotechnol Biochem 78:1183–1186. 10.1080/09168451.2014.885823 25229854 10.1080/09168451.2014.885823

[CR53] Sato S, Kitamoto D, Habe H (2014b) Chemical mutagenesis of *Gluconobacter frateurii* to construct methanol-resistant mutants showing glyceric acid production from methanol-containing glycerol. J Biosci Bioeng 117:197–199. 10.1016/j.jbiosc.2013.07.004 23916855 10.1016/j.jbiosc.2013.07.004

[CR54] Sato S, Kitamoto D, Habe H (2017) Preliminary evaluation of glyceric acid-producing ability of *Acidomonas methanolica* NBRC104435 from glycerol containing methanol. J Oleo Sci 66:653–658. 10.5650/jos.ess16236 28515381 10.5650/jos.ess16236

[CR55] Sauer T, Galinski EA (1998) Bacterial milking: a novel bioprocess for production of compatible solutes. Biotechnol Bioeng 57:306–313. 10.1002/(SICI)1097-0290(19980205)57:3%3c306::AID-BIT7%3e3.0.CO;2-L 10099207

[CR56] Sawangwan T, Goedl C, Nidetzky B (2009) Single-step enzymatic synthesis of ( R )-2-*O*-α-D-glucopyranosyl glycerate, a compatible solute from micro-organisms that functions as a protein stabiliser. Org Biomol Chem 7:4267–4270. 10.1039/B912621J 19795066 10.1039/b912621j

[CR57] Sawangwan T, Goedl C, Nidetzky B (2010) Glucosylglycerol and glucosylglycerate as enzyme stabilizers. Biotechnol J 5:187–191. 10.1002/biot.200900197 19946880 10.1002/biot.200900197

[CR58] Schwarz A, Goedl C, Minani A, Nidetzky B (2007) Trehalose phosphorylase from *Pleurotus ostreatus*: characterization and stabilization by covalent modification, and application for the synthesis of α, α-trehalose. J Biotechnol 129:140–150. 10.1016/j.jbiotec.2006.11.022 17222933 10.1016/j.jbiotec.2006.11.022

[CR59] Schwarz T (2005) Use of beta-mannosylglycerate and derivatives in cosmetic and dermatological formulations. U.S. Patent Application No. 10/344,971

[CR60] Sheldon RA, Woodley JM (2018) Role of biocatalysis in sustainable chemistry. Chem Rev 118:801–838. 10.1021/acs.chemrev.7b00203 28876904 10.1021/acs.chemrev.7b00203

[CR61] Suresh A, Shravan Ramgopal D, Panchamoorthy Gopinath K, Arun J, SundarRajan P, Bhatnagar A (2021) Recent advancements in the synthesis of novel thermostable biocatalysts and their applications in commercially important chemoenzymatic conversion processes. Bioresour Technol 323:124558. 10.1016/j.biortech.2020.124558 33383359 10.1016/j.biortech.2020.124558

[CR62] Taniguchi H, Sasaki T, Kitaoka M (1991) Method for preparing cellobiose. U.S. Patent No. 5,077,205. Washington, DC: U.S. Patent and Trademark Office

[CR63] Teng Y, Gu C, Chen Z, Jiang H, Xiong Y, Liu D, Xiao D (2022) Advances and applications of chiral resolution in pharmaceutical field. Chirality 34:1094–1119. 10.1002/chir.23453 35676772 10.1002/chir.23453

[CR64] Tsapis A, Kepes A (1977) Transient breakdown of the permeability barrier of the membrane of *Escherichia coli* upon hypoosmotic shock. Biochim Biophys Acta BBA - Biomembr 469:1–12. 10.1016/0005-2736(77)90320-0 10.1016/0005-2736(77)90320-0 329877

[CR65] van Hellemond EW, Vermote L, Koolen W, Sonke T, Zandvoort E, Heuts DPHM, Janssen DB, Fraaije MW (2009) Exploring the biocatalytic scope of alditol oxidase from *Streptomyces coelicolor*. Adv Synth Catal 351:1523–1530. 10.1002/adsc.200900176

[CR66] Villa A, Veith GM, Prati L (2010) Selective oxidation of glycerol under acidic conditions using gold catalysts. Angew Chem 122:4601–4604. 10.1002/ange.201000762 10.1002/anie.201000762 20461742

[CR67] Villa A, Dimitratos N, Chan-Thaw CE, Hammond C, Prati L, Hutchings GJ (2015) Glycerol oxidation using gold-containing catalysts. Acc Chem Res 48:1403–1412. 10.1021/ar500426g 25884231 10.1021/ar500426g

[CR68] Wada R, Hyon S-H, Ikada Y (1996) New biodegradable oligoesters for pharmaceutical application. J Biomater Sci Polym Ed 7:715–725. 10.1163/156856296X00480 8639480 10.1163/156856296x00480

[CR69] Wahart AJC, Staniland J, Miller GJ, Cosgrove SC (2022) Oxidase enzymes as sustainable oxidation catalysts. R Soc Open Sci 9:211572. 10.1098/rsos.211572 35242351 10.1098/rsos.211572 PMC8753158

[CR70] Winter RT, Heuts DPHM, Rijpkema EMA, van Bloois E, Wijma HJ, Fraaije MW (2012) Hot or not? Discovery and characterization of a thermostable alditol oxidase from *Acidothermus cellulolyticus* 11B. Appl Microbiol Biotechnol 95:389–403. 10.1007/s00253-011-3750-0 22231860 10.1007/s00253-011-3750-0 PMC3371188

[CR71] Yakushi T, Matsushita K (2010) Alcohol dehydrogenase of acetic acid bacteria: structure, mode of action, and applications in biotechnology. Appl Microbiol Biotechnol 86:1257–1265. 10.1007/s00253-010-2529-z 20306188 10.1007/s00253-010-2529-z

[CR72] Zhang T, Yang J, Tian C, Ren C, Chen P, Men Y, Sun Y (2020) High-yield biosynthesis of glucosylglycerol through coupling phosphorolysis and transglycosylation reactions. J Agric Food Chem 68:15249–15256. 10.1021/acs.jafc.0c04851 33306378 10.1021/acs.jafc.0c04851

[CR73] Zhang C, Chen Q, Fan F, Tang J, Zhan T, Wang H, Zhang X (2021) Directed evolution of alditol oxidase for the production of optically pure D-glycerate from glycerol in the engineered *Escherichia coli*. J Ind Microbiol Biotechnol 48:kuab041. 10.1093/jimb/kuab041 10.1093/jimb/kuab041 PMC8788829 34196357

